# The Association Between β-Dystroglycan in Airway Smooth Muscle and Eosinophils in Allergic Asthma

**DOI:** 10.1007/s10753-020-01401-y

**Published:** 2021-02-10

**Authors:** Suhayla H. Shareef, Kawa Amin, Christer Janson

**Affiliations:** 1 Department of Microbiology/Immunology, College of Medicine, University of Sulaimani, Sulaymaniyah, Iraq; 2 Department of Medical Science, Respiratory Medicine, and Allergology, Uppsala University and University Hospital, Uppsala, Sweden

**Keywords:** allergic asthma, airway smooth muscle, β-dystroglycan, eosinophil, inflammation, remodeling

## Abstract

Allergic asthma (AA) is a complex disorder with heterogeneous features of airway hyperresponsiveness, inflammation, and remodeling. The increase of airway smooth muscle (ASM) mass is a fundamental component of bronchial remodeling in AA, yet the pathophysiological mechanisms and clinical outcomes associated with ASM modulation are still elusive. The objective of this study is to compare the expression level of β-dystroglycan (β-DG) in ASM in AA subjects and a healthy control group and to investigate the relationship between eosinophils and β-DG in ASM in patients with AA. Thirteen AA patients and seven control subjects were analyzed for the ASM area and eosinophil cells. Bronchial biopsies were stained by β-DG and eosinophil cationic protein (ECP) using immunohistochemistry. The proportion of ASM with β-DG staining was greater in those with AA than in the healthy control group (mean (95% CI) (28.3% (23.8–32.7%) *vs.* 16.4% (14.1–18.5%), *P* < 0.0001). The number of ECP positive cells was higher in patients with AA than in the control group (4056 (3819–4296) *vs.* 466 (395–537) cells/mm^2^ *P* < 0.0001). In AA, the number of ECP positive cells was significantly correlated to the β-DG expression in ASM (*r* = 0.77, *P* = 0.002). There is an increased β-DG expression in ASM and a higher number of ECP positive cells in the bronchial biopsy of those with AA than those in the control group. The increased expression of β-DG in ASM in AA subjects correlates with the number of eosinophils, suggesting a role for this cell in airway remodeling in AA.

## INTRODUCTION

Allergic asthma (AA) is a heterogeneous and chronic inflammatory disorder associated with airway hyperresponsiveness (AHR), airway remodeling, and subsequently declining airway function [1]. It is characterized by a history of variable and repetitive respiratory symptoms that vary over time and in intensity, reversible airflow obstruction, and bronchospasm [2].

The chronic inflammation of AA is accompanied by the infiltration of multiple proinflammatory cells, such as eosinophils, lymphocytes, mast cells, and mediators [3], and involves both the innate and adaptive pathways of the immune system [4]. The hallmarks of airway remodeling in asthma comprise structural modifications in the bronchial wall: epithelial damage, subepithelial fibrosis, goblet cell hyperplasia/enlargement, mucus plugging, smooth muscle cell hyperplasia and/or hypertrophy, excessive extracellular matrix production [5], and angiogenesis of the bronchial vasculature [6]. There is, however, considerable heterogeneity in the inflammatory patterns between different types of asthma [7].

Airway smooth muscle (ASM) tissues are specially designed to stiffen, shorten, or relax, thereby organizing the diameter of the bronchi and/or bronchioles they encircle [8]. ASM plays a key role in the pathogenesis of asthma by undergoing evident phenotypic changes, implicating the acquisition of a hypercontractile, hyperresponsive, and profibrotic state [9]. Moreover, a strong relationship exists between phenotypic changes and airway function in ASM. For instance, AHR, a fundamental feature of asthma, is defined as the exaggerated airway narrowing to constrictive stimuli, which is highly associated with ASM hypercontractility [10]. Despite its contractile activity, airway myocytes can also be motivated to migrate, proliferate, and secrete extracellular matrix (ECM), growth factors (GF), cytokines, and chemokines, thereby contributing to fibroproliferative remodeling of the airway walls during the pathogenesis of asthma [11]. Likewise, the multifunctional behavior of airway myocytes stems from a capability for reversible phenotype switching between contractile and proliferative/synthetic states [11]. The expression of smooth muscle proteins can be used as a marker for myocyte function in bronchial biopsies taken from the airways, vasculature, or other hollow organs [12].

ASM mass excess is an important aspect of both AHR and airway remodeling [13]. ASM mass accumulation can be triggered by multiple factors, including inflammation and bronchoconstriction. There is an ongoing debate regarding the relative contributions of ASM cell proliferation, hypertrophy, and recruitment (for example, *via* differentiated fibroblasts and myofibroblasts) in asthmatic airways [14]. A large number of inflammatory mediators are associated with ASM proliferation *in vitro*, and infiltrating inflammatory cells are a feature of asthma [15]. In a murine model, hypertrophic ASM mass correlates with increased airway responsiveness [16]. In a guinea pig model, ASM hyperreactivity was induced by chronic allergic lung inflammation [17]. The role of inflammation resolution speed in ASM mass accumulation in asthma has been studied using a non-invasive theoretical model [18].

Dystroglycan (DG) represents a core component of the dystrophin-glycoprotein complex (DGC), which is an integral membrane receptor that anchors an intracellular actin-based cytoskeleton to extracellular laminin [19]. DG is coded by a single gene (DAG1) and translated for a polypeptide precursor that undergoes post-translational proteolytic cleavage to generate two mature noncovalent subunits (α and β) DG [19]. α-DG is a dumbbell-shaped peripheral membrane protein that interacts with the laminin G domain in the ECM proteins [19], while β-DG is a 43 kDa type 1 transmembrane glycoprotein that anchors to the carboxy-terminal domain of α-DG on the extracellular side. Its cytosolic domain anchors to the actin cytoskeleton through association with dystrophin and other cytolinker proteins [19, 20]. Besides a primarily structural role in the maintenance of the sarcolemmal stability, the DGC has been shown to participate in other cellular processes, including mechanotransduction, Ca^2+^ homeostasis, and tissue morphogenesis [20, 21]. β-DG particularly modulates a plethora of cellular functions, acting as a platform for cytoskeleton remodeling and cell adhesion systems in various cell types and tissues [20]. However, there has been little research into the β-DG molecule in human pathology despite it being considered a functionally significant protein since it holds the cytoplasmic structural and signaling region [22].

Eosinophilic inflammation is a characteristic phenotype of asthma with an overflow of eosinophils in the bronchial airways [23]. Eosinophilic inflammation in asthma emerges from intricate immunologic and proinflammatory mechanisms, fundamentally orchestrated by allergic sensitization and T helper (Th)2 lymphocytes [24]. The eosinophil-derived transforming growth factor-β (TGF-β) activates a fibroblast release of matrix proteins and increases ASM mass, thereby promoting airway remodeling and the development of subepithelial fibrosis [25, 26]. Eosinophil cationic protein (ECP) is used as a clinical marker for eosinophil activity in asthma.

To our knowledge, there are no investigations into the expression levels of β-DG in the ASM of patients with AA and its relationship with eosinophils. We hypothesized that in AA, the immunolocalization of activated eosinophil and β-DG within SM tissues is overexpressed compared to that in the airway of the control group. The aim of this study was, therefore, to compare the expression of β-DG in ASM between subjects with AA and a healthy control group and to investigate the relationship between eosinophil and β-DG expression in ASM in AA.

## MATERIALS AND METHODS

### Subjects

Bronchial biopsies were taken from 20 non-smoking adults who were split into two groups: patients with mild and moderate stable AA (*n* = 13) and healthy control subjects (*n* = 7). The clinical and demographical characteristics of the patients and the control group are presented in Table 1. The participants were part of a larger group from an ongoing study of the pathophysiology of AHR [28]. All AA patients had a clinical diagnosis of asthma, existing asthma symptoms, and increased responsiveness to inhaled methacholine, defined as the provocative concentration of methacholine causing a ≥ 20% reduction in FEV1 (PC20 ≤ 32 mg) (Table 1). The participants in the AA group were non-smokers, had not had any clinical features of infection for at least 4 weeks before starting the study, and had no history of cardiovascular disease. All subjects with AA had positive skin prick tests (SPTs) (wheal size ≥3 mm) for one or more of the aeroallergens. None in the control group had asthmatic symptoms or was receiving anti-asthmatic treatment, and all had negative SPTs. Furthermore, those AA participants sensitive to pollens were studied outside of the pollen season. A symptom diary was kept during a 17-day period in which the subjects stated whether they had breathing difficulties during the previous night, wheezing in the chest, attacks of breathlessness, or attacks of coughing during the previous 24 h. Each affirmative answer was given a score of one, and a symptom score was calculated.

**Table 1:** Patient Characteristics (Mean [Range] and *n*) [27]

	Healthy controls (*n* = 7)	Allergic asthma (*n* = 13)
Age (year)	30 (22–44)	38 (19–63)
Sex (women)	5	9
Inhaled corticosteroids^#^	0	9
FEV_1_ (% pred)	98 (77–120)	96 (72–132)
FVC (% pred)	95 (78–109)	104 (86–141)
Symptom score	0.1	2.4 (0–4)
PEF variability (%)	5 (3–9)	11 (5–22)
PC_20_ (mg/ml)	-	6.1 (0.07–32)

Definition of abbreviations: *FEV1*, forced expiratory volume in 1; *FVC*, forced vital capacity; *PEF*, peak expiratory flow; *PC20*, provocation concentration that reduces FEV1 by 20%

^#^Currently using

### Procedure

Fiber bronchoscopy was performed according to a previously described protocol [27].

### Preparation of Frozen Tissues

For immunohistochemical analysis of a certain antigen expression, freshly obtained bronchial biopsy specimens were immediately frozen in phosphate-buffered saline (PBS) and melted later in isopentane, previously cooled in liquid nitrogen. Frozen biopsies were preserved in liquid nitrogen until being sectioned. The sections were attached to the specimen holder of a cryostat microtome (Microm, HM 500 M; Heidelberg, Germany) in a drop of optimum cutting temperature (OCT) compound (Tissue-Tek; Miles, Elkhart, IN), and the tissue was cryosectioned serially into 5-μm-thick slices. The sections were collected by touching the section set on the knife with a warm microscope slide. After drying the sections at room temperature, they were rolled in aluminum foil and stored at − 80 °C until they were used for immunohistochemistry.

### Immunohistochemistry

The horseradish peroxidase (HRP) method was used to identify the specific binding of C-term β-DG protein at cytosol on bronchial SM cells. The HRP reaction has visualized the distribution and localization of specific intracellular β-DG components by using the appropriate 3,3 N-diaminobenzidine tetrahydrochloride (DAB) kit. A rabbit polyclonal dystrophin-associated glycoprotein 1 (DAG1)/beta-dystroglycan (β-DG) antibody (C2C3-2) C-term (Gene Tex system, USA, GTX124225) was used for the identification of human β-DG protein, and the monoclonal EG2 antibody (eosinophil cationic protein/eosinophil protein X; ECP/EPX) (Pharmacia Upjohn, Diagnostics AB) was used to identify eosinophil on 5 μm frozen bronchial sections. The cryostat sections were thawed, then fixed with acetone at − 20 °C for 15 min at room temperature before incubation with the primary antibodies. Tissue sections were incubated in 10% normal rabbit serum with a blocking agent to minimize non-specific background staining for 20 min. The primary antibodies of rabbit anti-human β-DG polyclonal and ECP monoclonal antibodies were added after they were diluted in PBS (1X). Antibodies against β-DG and ECP were diluted to 0.02 μg/ml and 0.03 μg/ml, respectively, in Tris-HCl buffer (0.01 mol/l, pH 7.4) and incubated at 4 °C for 3 h in a humid chamber. The sections were washed three times with Tris-HCl buffer, followed by incubation with an appropriate biotinylated secondary antibody (HRP-Linked Guinea pig Anti-Rabbit IgG Polyclonal Antibody) (Cloud-Clone Corp, USA, SAA544Rb59). The secondary antibody was diluted in the blocking buffer at room temperature. The incubation lasted for 1 h and the section was washed three times with PBS at room temperature for 5 min. The antigen-antibody complex was imaged using commercial diaminobenzidine (DAB) Kit (Cloud-Clone Corp, USA, IS046) as one of the most essential chromogens for peroxidase, according to the manufacturer’s instructions to produce a brown end product. In the negative controls, there was no expression on the surface of the sections of the primary antibodies. After washing, the samples were counterstained with Mayer’s hematoxylin (Merck D-6071, Darmstadt, Germany) for 6 min and, finally, the cover glasses were mounted using a Faramount Aqueous Mounting Medium (Dako, USA, S3025).

### Quantification of β-DG and the ECP Positive Cell

The variation in cell counts and estimates of structural changes between the two microscopic sections varied between 3 and 5% (% coefficient of variation). All the specimens were coded and examined by the microscopist without knowledge of the diagnosis. The number of coded cells in the tissue was enumerated manually at a magnification of × 100 and the measurement of the ASM thickness in both the healthy control and AA group was performed according to a previously described protocol [29]. The number of sections that could be obtained in a particular biopsy was not sufficient in all cases for incubation with all the antibodies. Before the use of the β-dystroglycan antibody for identifying smooth muscle, actin smooth muscle antibody was used to detect the smooth muscle (data not shown). All the IHC processes used manual inspections. The total of the ASM area stained with β-dystroglycan was divided into the whole (with and without stain) ASM area in biopsy sections and multiples of 100. The area was measured using a Leica DMLB microscope for image analysis (Wetzlar GmbH, Germany) equipped with a Leica Microsystem digital camera (DC 300F) connected to a computer. The images were captured and saved on the computer for further evaluation using the software package Qwin v2.7 after calibration with the aid of a stage squared micrometer. In each biopsy, two subsequent sections were evaluated.

Eosinophil was identified with monoclonal antibodies (EG2) against their granule proteins. The extracellular deposition of eosinophil products and non-extracellular deposition of eosinophil products was identified in the lamina propria and SM. Eosinophils were categorized as eosinophils with extracellular deposition of eosinophil products when extracellular deposition of anti-ECP stained material was observed [30].

### Statistical Analysis

The statistics were calculated using non-parametric tests. Comparisons between AA and healthy subjects were performed using the Mann–Whitney *U*-test. The Spearman’s rank correlation coefficient was used to quantify the relationship between the β-DG and ECP in bronchial biopsy sections. All analyses were fulfilled using Prism 7.0 Software (Graphpad, La Jolla, California, USA). *P* < 0.05 was deemed significant.

## RESULTS

### A Comparison of β-DG Between the AA and Healthy Control Groups

**Fig. 1:**
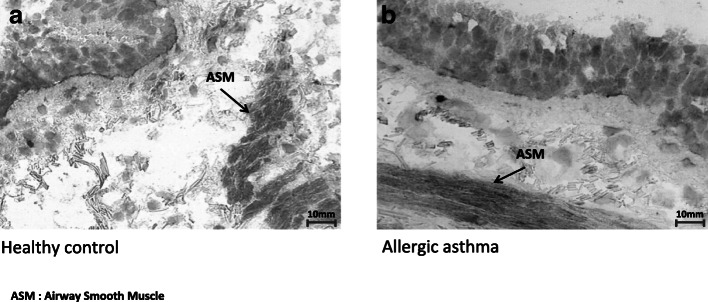
Immunohistochemical analysis of the human bronchial biopsy specimens. **a** Expression of the β-DG subunit in human ASM tissue from the healthy control group. **b** β-DG subunit in human ASM tissue from the AA group. The frozen biopsies were cryosectioned serially into thin slices (5 μm) and then subjected to immunostaining by DAB and light microscopy examination of the thickness of the ASM by detection of the C-terminal β-DG protein at cytosol in ASM. Note the strong expression of β-DG in the muscle fiber membranes (arrows). Note also the poor/moderate staining in the SM cell layer (arrows). The nuclei were counterstained with Mayer’s hematoxylin. The location of the ASM layer and epithelium are indicated in the primary antibody stained panels as the brown color. The original magnification is × 40. The scale bars are 10 μm. EOS, eosinophil cells; ASM, airway smooth muscle.

In the current study, we first addressed the question of whether the β-DG subunit is present in ASM tissue by assessing immunoreactivity with immunohistochemical analysis and whether β-DG is down or overexpressed in AA compared to that in the healthy controls. Figure 1 shows that immunolabeling produced by C-terminal β-DG antibodies was predominantly localized to the cytoplasm and plasma membrane of SM cells. Immunohistochemical images revealed higher β-DG expression in ASM in patients with AA than in the healthy control group (Fig. 1, a and b).

**Fig. 2:**
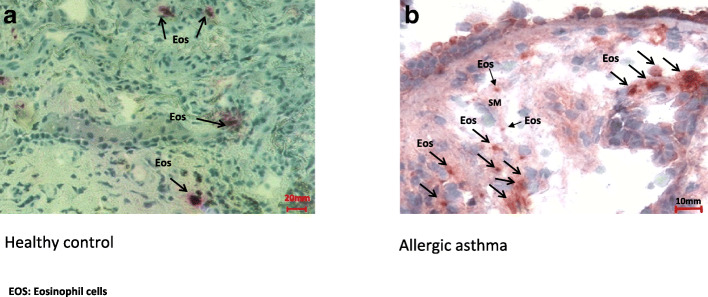
Immunocytochemical staining of bronchial biopsy specimens for ECP. Eosinophil cells are visualized using a monoclonal antibody (EG2) detecting the granule protein ECP. In healthy controls (**a**), eosinophils are staining weakly for ECP (scale bars = 20 μm), original magnification (× 20). In contrast to AA (**b**), a high number of eosinophils and high release activity of ECP can be found between the epithelial cells and in the submucosa with the areas of epithelial damage and reduced epithelial integrity (arrows, scale bar = 10 μm). Eosinophils were expressed close to ASM cells (arrow). Original magnification (× 40); nuclei were counterstained with Mayer’s hematoxylin. EOS, eosinophil cells.

**Fig. 3:**
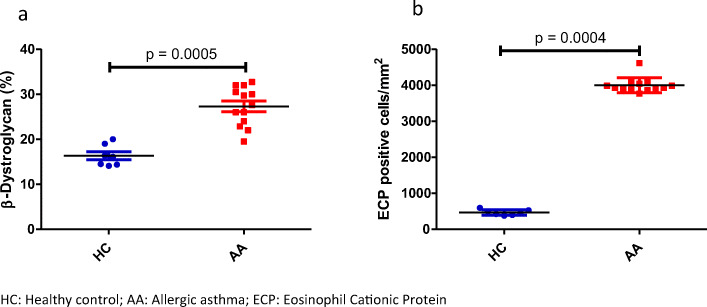
Statistical analysis for the comparison of the thickness of β-DG in patients with AA (**a**) and ECP positive cells (**b**) in healthy controls. A highly significant difference between the groups (*P* < 0.01) was shown using the Mann–Whitney test and graph pad software. HC, healthy control; AA, allergic asthma; ECP, eosinophil cationic protein.

The number of eosinophils was higher in the tissue of patients with AA than in the healthy control group (Fig. 2, a and b). Statistical analysis demonstrated a significantly higher expression of β-DG in ASM (a) and ECP positive cells (b) (mean (95% CI) 28.3% (23.8–32.7%) *vs.* 16.4% (14.1–18.5%)), (95% CI) 11.9% (7.2–14.6%), and 95% CI for ECP in AA 4058 (3819–4296) *vs.* 466 (395–537) cells/mm^2^ (3592 (3392–3664) cells/mm^2^, respectively (*P* < 0.001) (Fig. 3).

The mean ASM area was larger in the group with AA than in the control group (mean (95% CI): 72.7% (70.1–75.3%) *vs.* 16.35% (14.2–18.5%), (95% CI) 44.53% (42.15–46.9%), *P* = 0.0004).

### Correlation Between the β-DG and Eosinophil Cells

**Fig. 4:**
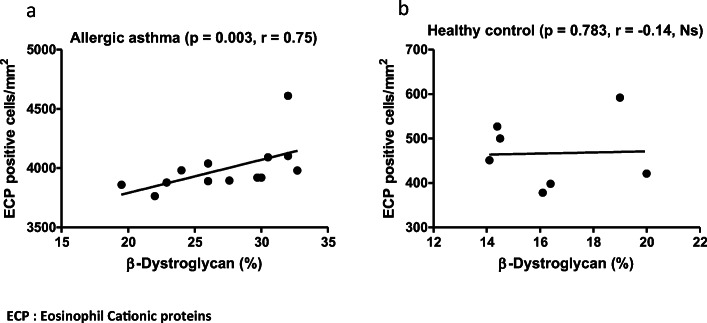
Scatter plot demonstrating the positive correlation between the number of ECP positive cells and β-DG thickness in AA (*P* < 0.01). No significant correlation was found between ECP positive cells and β-DG in the healthy control group. ECP, eosinophil cationic proteins.

In the AA group, a significant positive correlation was found between the expression of β-DG in ASM and the number of eosinophil cells (*r* = 0.77, *P* = 0.002) (Fig. 4). There was no correlation between β-DG and ECP positive cells (*r* = − 0.14, *P* = 0.783) in the control group.

## DISCUSSION

The study reveals a higher expression of β-DG in ASM in patients with AA than in the control group. An association has been found between β-DG in ASM and eosinophils in AA. The proportion of ASM with β-DG staining was greater in AA than in the healthy controls. Limited data exist regarding the role of DG proteins in airway remodeling in asthma patients. To the best of our knowledge, the current study is the first to compare the expression of β-DG in ASM in patients with AA and with a healthy control group. The study indicates that β-DG has an important role in ASM remodeling and provides the first indication of its role in lung physiology of AA disease.

As described in numerous studies, β-DG is the core membrane–spanning component of the DGC known as a dystrophin-associated transmembrane protein involved in plasma membrane integrity, stability, adhesion, signal transduction, and cytoskeletal remodeling [31, 32]. β-DG is a widely expressed protein in various cell types and tissues that connects the intracellular cytoskeleton to the extracellular matrix [33] and plays important biological roles in both development and disease [34]. Both an overexpression and depletion of β-DG affects the size and number of different adhesion types. The reduction of DG increases the number of adhesions while a high expression decreases their size and number [35]. We suggest that the maturation of fibrillar adhesions relies on the expression level of DG because overexpressing or reducing levels of overall DG proteins affects the number and size of these adhesions. For this reason, an increase in the number or size of fibrillar adhesions would be expected to influence cell motility. Therefore, the overexpression of β-DG in ASM cells in AA may lead to a reduction in fibrillar adhesions that are associated with an increase in velocity and a decrease in stability and loss of polarity, which consequently decreases the directed migration.

Previous work indicates that β-DG30 levels are raised in the striated muscle of cardiomyopathic hamsters and also in the skeletal muscles of patients with Duchenne muscular dystrophy [36]. In a study into osteoarthritic synovium disease [37, 38], increased immunolocalization of β-DG in osteoarthritic synovium vascular endothelium was found, indicating that overexpression of β-DG may be related to a pro-angiogenic molecule and increased neovascularization in blood vessel density. In a study of oral squamous cell carcinoma [39], there was no β-DG expression in lymph node metastases despite the presence of β-DG in the corresponding primary tumors. Previous work by Sgambato et al. [40] reported a heterogeneous and low expression of DG related to a higher tumor stage, high proliferation index, and lower overall survival in the large series of breast and colon tumors.

In our study, we have shown that the number of eosinophils was higher in patients with AA than in the control group. We employed the specific monoclonal antibody EG2 to identify intracellular staining for ECP in eosinophil. EG2 has been shown to bind to ECP only during degranulation, recognizing eosinophils with more certainty than is possible to gather with purely morphological techniques [41]. Hurst and Venge [42] confirmed that EG2 is a monoclonal antibody for phenotyping ECP. It is well known that the bronchial tissue of AA is infiltrated with diverse types of inflammatory cells, including eosinophils, neutrophils, mast cells, and T lymphocytes. Eosinophil accumulation in the bronchial wall and lumen is a noticeable feature of asthma. Most of the available evidence from *in vivo* models suggests that eosinophils contribute directly to mucus hypersecretion, epithelial damage, AHR, remodeling, and airway dysfunction [39]. ECP has been observed to influence some cell types present in the respiratory tract. Accumulation and infiltration with eosinophils (EG2^+^) have been observed in bronchial biopsies of asthma, rhinitis, and cutaneous responses after an antigen challenge in atopic individuals [43].

In the present study, an association has been found between β-DG in ASM and eosinophils in AA. This indicates that eosinophils play a role in airway remodeling in AA. Airway remodeling is described as an increased thickening of the bronchial wall due to various structural alterations of ASM membrane thickening. The persistently high number of eosinophils and ECP in the airways of patients with AA may lead to architectural remodeling by enhancing the proliferation of ASM cells [44]. Halwani et al. [45] confirmed that preventing eosinophil adhesion with ASM cells using specific blockers or antibodies to cysteinyl leukotriene derived from eosinophil is related to the inhibition of ASM proliferation. ASM-synthesized cytokines also appear to directly affect eosinophil recruitment, differentiation, and maturation from progenitor cells [46]. Experimental studies have shown that ECP can activate the calcium-sensing receptor on ASM cells. Asthmatic patients and allergen-sensitized mice expressed a high level of this receptor [47].

A limitation of our study is that we only included a limited number of participants. Furthermore, we lack data from patients with severe asthma and patients with non-allergic asthma.

## CONCLUSIONS

We found an increased β-DG expression in ASM and a high number of ECP in the bronchial biopsy of AA subjects than in the control group. The increased expression of β-DG in ASM in AA subjects correlates with the number of eosinophil cells, suggesting a role for this cell in airway remodeling of the AA.

## Data Availability

Not applicable.
